# Temperature stressed *Caenorhabditis elegans* males fail to mate successfully and successful males produce very few viable cross progeny

**DOI:** 10.17912/micropub.biology.000475

**Published:** 2021-10-19

**Authors:** Nicholas B Sepulveda, Lisa N Petrella

**Affiliations:** 1 Marquette University

## Abstract

Exposure to moderate temperature stress can have profoundly negative effects on an organism’s reproductive capacity at temperatures where there are minimal or indiscernible effects on the organism as a whole. These negative effects are often more pronounced in males of the species that produce sperm. Previously we showed that few males of *Caenorhabditis elegans* wild type strains are able to successfully produce any cross progeny after experiencing temperature stress. However, these experiments did not assess the number of progeny from temperature stressed males. To understand if temperature stress can reduce the number of progeny a male sires, we crossed temperature stressed males of three wild type strains of *C. elegans*: JU1171, LKC34, and N2, to strain matched hermaphrodites of their own genetic background or to uncoordinated hermaphrodites in the N2 background. We found that significantly fewer males exposed to moderate temperature stress can successfully mate and that the small number of males in the population that do successfully mate produce significantly fewer viable cross progeny than unstressed controls. Our results suggest that exposure to moderate temperature stress significantly reduces male *C. elegans* chances at reproducing similar to what is seen in other organisms.

**Figure 1. JU1171, LKC34, and N2 males were less likely to mate successfully and produced significantly fewer viable cross progeny at 27°C:**
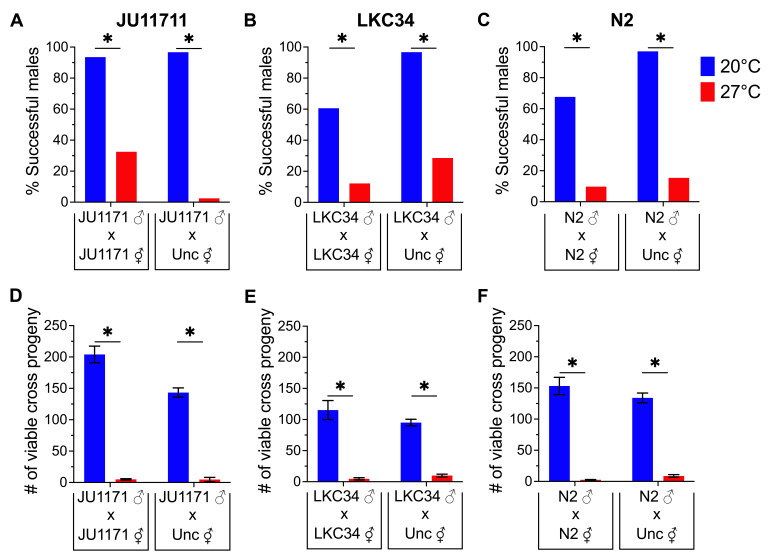
Males were either raised at 20°C (blue bars) or upshifted to 27°C at L1 stage (red bars) and crossed with either strain matched or *unc-24(e138*) (Unc) hermaphrodites. (A-C)The percentage of males that mated successfully and produced viable cross progeny (% Successful males) was assessed. Significantly fewer males of all three wild type strains mated successfully to produce viable cross progeny at 27°C versus 20°C (Fisher’s Exact Test, *P < 0.05). (D-F) The number of viable cross progeny produced by successful males was assessed (dead eggs and unfertilized oocytes were not counted). Males of all three wild type strains produced significantly fewer viable cross progeny at 27°C versus 20°C (Two-way ANOVA with Tukey’s correction, *P < 0.05). Error bars represent ± 1 SEM.

## Description

Like most species, *Caenorhabditis elegans* show a dramatic decrease in fertility when exposed to increasing temperatures (Harvey and Viney, 2007; Petrella, 2014; Poullet *et al.*, 2015). In hermaphrodites, the susceptibility of sperm to temperature stress is the foremost factor defining the decline in fertility observed in several wild type strains of the species (Harvey and Viney, 2007; Petrella, 2014; Poullet *et al.*, 2015). While some studies have looked directly at the impact of temperature stress on the males of the species (Nett *et al.*, 2019; Petrella, 2014; Poullet *et al.*, 2015), the conflation of temperature effects on male mating behavior, mating success, and male sperm function have made these studies more difficult. Previously, we showed that *C. elegans* males of several wild type strains have low mating success when exposed to moderate temperature stress (27°C), as measured by the percentage of males that had any viable cross progeny when crossed with *unc-119* hermaphrodites (Petrella, 2014). However, we did not assess if those males that show some level of fertility had a decrease in the number of viable cross progeny that they could produce, as is seen in hermaphrodites exposed to moderate temperature stress (Harvey and Viney, 2007; Petrella, 2014; Poullet *et al.*, 2015). These previous experiments were also done with males of each wild type strain crossed with the *unc-119* mutant hermaphrodites in the N2 background, thus genetic incompatibilities could not be ruled out as playing a role in the decreased mating success. To avoid genetic incompatibilities and assess male fertility in a way that would more accurately recapitulate the most likely scenario in the natural habitat of *C. elegans* (Barrière and Félix, 2005; Richaud *et al.*, 2018), we crossed males of three wild type strains of *C. elegans*: JU1171, LKC34, and N2 with strain matched wild type hermaphrodites. As genetic incompatibility may not be a strong factor, we also crossed wild type males with *unc-24(e138*) hermaphrodites in the N2 background because Unc hermaphrodites have limited motility, which would facilitate higher male mating success. Thus, we may be more likely to capture any viable progeny that could be produced by males of each strain because they are more likely to successfully mate.

We found that significantly fewer temperature stressed males of all three wild type strains were able to successfully mate at 27°C versus their unstressed counterparts at 20°C, whether crossed with strain matched wild type hermaphrodites or *unc-24(e138*) hermaphrodites (Figure 1A-C). In addition, the temperature stressed males that do achieve mating success at 27°C produced significantly fewer viable cross progeny versus their unstressed counterparts at 20°C, whether crossed with strain matched wild type hermaphrodites (hermaphrodites of their own strain) or *unc-24(e138*) hermaphrodites (Figure 1D-F). As we did not assess dead eggs or unfertilized oocytes this may be due to either an increase in embryonic lethality or fewer viable sperm being made or transferred. However, growth at stressful temperatures overall leads to a reduced number of viable cross progeny even when a male successfully mates with a hermaphrodite.

We predicted that more males from each wild type strain would successfully mate when crossed with *unc-24(e138*) hermaphrodites because these hermaphrodites have impaired motility and cannot exert mate choice behavior (Bahrami and Zhang, 2013). We found a significant increase only in the percentage of males that mated successfully with *unc-24(e138)* hermaphrodites versus strain matched hermaphrodites for N2 at 20°C and LKC34 at both 20°C and 27°C (Figure 1B-C). For both N2 and LKC34 there was no difference in the number viable progeny sired by those males (Figure 1E-F). This suggests that LKC34 hermaphrodites likely exercise mate choice as has been previously shown for N2 (Bahrami and Zhang, 2013) at both temperatures. However, hermaphrodite mate choice may be impacted by temperature stress in N2 hermaphrodites, such that N2 males are equally successful with strain matched or *unc-24(e138*) hermaphrodites at 27°C. Interestingly, there was no change in the percentage of JU1171 males that mated successfully with *unc-24(e138)* hermaphrodites versus strain matched hermaphrodites for JU1171 at 20°C, and there was a significant decrease in the number of males that could mate successfully at 27°C (Figure 1A). In addition, we saw a significant decrease in the number of viable cross progeny from JU1171 males at 20°C when crossed to *unc-24(e138*) hermaphrodites versus strain matched hermaphrodites (Figure 1D). These differences are likely due to a JU1171-N2 genetic incompatibility, which has been observed previously, and shown to limit viable progeny from this specific genetic cross (Seidel *et al.*, 2008).

These data suggest that exposure to moderate temperature stress has a large and significant effect on the percentage of *C. elegans* males that can achieve mating success and the number of viable cross progeny they sire. In the context of climate change associated elevations in global temperature, our results underscore the overwhelming reproductive challenges faced by susceptible populations of sexually reproducing organisms.

## Methods

**Worm maintenance and temperature treatment**

*Caenorhabditis elegans* worms were maintained using standard methods (Brenner, 1974) at 20°C on nematode growth media (NGM) plates spotted with AMA1004 *Escherichia coli*. Males of the three wild type strains JU1171, LKC34, and N2 were generated by heat shocking L4 hermaphrodites at 30°C for five hours followed by recovery at 20°C and selection of male progeny. Male populations were subsequently maintained by crossing males and hermaphrodites of the same genotype at 20°C. The wild type strains used in this study were originally isolated from the following localities: JU1171 (Concepción, Chile) (Dolgin *et al.*, 2008), LKC34 (Madagascar) (Dolgin *et al.*, 2008), and N2 (Bristol, UK) (Brenner, 1974). Two temperature treatments were used in this study. Continuous exposure to 20°C: Male and hermaphrodite worms were maintained continuously at 20°C. Continuous exposure to 27°C: Male worms were upshifted from 20°C to 27°C at the L1 stage and maintained continuously at 27°C until assayed, and hermaphrodite worms were maintained at 20°C until the L4 stage.

**Viable cross progeny counts and assessment of male mating success**

One day before the experiment NGM test plates were prepared by spotting them with 50 ml AMA1004 *E. coli* grown to OD_600_ = 1.0. On the day of the experiment, a single L4 male and L4 hermaphrodite were placed on a test plate. Males were crossed with a hermaphrodite of the same strain or with a CB138 [*unc-24(e138)*] hermaphrodite and placed at the assay temperature. Males and hermaphrodites were allowed to mature to adulthood, mate, and produce progeny for 24 hours. Subsequently, male worms were destroyed, and hermaphrodites were moved to a new NGM plate. Hermaphrodites were allowed to produce progeny for an additional 24 hours before they were destroyed. F1 progeny were allowed to mature to at least the L3 stage before counting. Only viable progeny were counted, dead eggs and unfertilized oocytes were not assessed. F1 male and hermaphrodite progeny were scored by picking individual worms and destroying them. For 20°C experiments, all male progeny were considered to be true viable cross progeny that came from male sperm. To calculate the total number of viable cross progeny, the number of male progeny was doubled to approximate hermaphrodite cross progeny. For 20°C experiments, males were considered successful at mating if at least one male progeny was produced from a cross. At 27°C we have observed that hermaphrodites have male progeny at a higher rate than at 20°C. Therefore, when assessing viable cross progeny and mating success for 27°C experiments, the increase in spontaneous male progeny by hermaphrodites was considered. For these experiments, crosses that yielded two or fewer males were considered to have only viable self-progeny produced from hermaphrodite sperm. For each experiment, a minimum of six crosses were assayed (n ³ 6) per strain and temperature treatment, and at least five biological replicates per strain and temperature treatment were performed in total (n ³ 30). Statistical analyses were conducted using Prism 9 (GraphPad, San Diego, CA, USA).

## Reagents

*C. elegans* strains were obtained from the *Caenorhabditis* Genetics Center (CGC) (Minneapolis, MN, USA) which is funded by NIH Office of Research Infrastructure Programs (P40 OD010440).

**Table:** 

**Strain**	**Genotype**	**Genetic** Background	**Available from**
JU1171	Wild type	JU1171	CGC
LKC34	Wild type	LKC34	CGC
CB138	*unc-24(e138*) IV	N2	CGC
N2	Wild type	N2	CGC
